# Reduced global BOLD-CSF coupling in chronic kidney disease-related cognitive impairment: a resting-state functional MRI study

**DOI:** 10.3389/fneur.2025.1738198

**Published:** 2026-01-12

**Authors:** Bingkui Yang, Feng Cui, Kexin Li, Yuxuan Zhu, Yu Zhang, Luping Zhang, Zhiqiang Yan, Ping Jin

**Affiliations:** 1 Department of Radiology, Hangzhou Traditional Chinese Medicine Hospital Affiliated to Zhejiang Chinese Medical University, Hangzhou, China; 2 Zhejiang Chinese Medical University, Hangzhou, China

**Keywords:** cerebrospinal fluid, chronic kidney disease, cognitive impairment, global BOLD signal, glymphatic system, kidney-brain axis, resting-state functional MRI

## Abstract

**Introduction:**

Cognitive impairment is a common complication of chronic kidney disease (CKD), but its underlying mechanisms are not fully understood. This study aims to investigate the glymphatic system function in CKD patients with and without cognitive impairment (CI) by analyzing the coupling between the global blood oxygen level-dependent (gBOLD) signal and the cerebrospinal fluid (CSF) signal using resting-state functional magnetic resonance imaging (rs-fMRI).

**Methods:**

Twenty-nine patients with CKD were enrolled (19 with CI and 10 without), along with 22 healthy controls (HCs). All patients underwent high-resolution structural MRI and rs-fMRI scans. The gBOLD–CSF coupling was quantified by calculating the maximum negative correlation within a predefined time-lag range between the gBOLD signal and the fourth ventricular CSF signal. The gBOLD-CSF coupling was compared between the CKD and HC groups using analysis of covariance (ANCOVA), adjusting for age, sex, education, and mean framewise displacement (FD). The difference between patients with CKD with and without CI was assessed using ANCOVA, after adjusting for age, sex, education, hypertension, diabetes, and mean FD. Partial correlation analysis was performed to explore the associations between gBOLD-CSF coupling and clinical indicators, such as estimated glomerular filtration rate (eGFR), Montreal Cognitive Assessment (MoCA) scores, and other laboratory data.

**Results:**

After adjusting for covariates, gBOLD-CSF coupling was significantly lower in the CKD group than in the HC group (*β* = −0.178, *p* = 0.003). This finding remained robust in sensitivity analyses adjusting for hypertension and diabetes. Within the CKD group, patients with CI had significantly lower gBOLD-CSF coupling than those without CI (*β* = −0.135, *p* = 0.040). Correlation analyses revealed that gBOLD-CSF coupling tended to be positively associated with hemoglobin, MoCA score, and eGFR, and negatively associated with blood urea and creatinine; however, none of these correlations reached statistical significance after false discovery rate correction (all *q* > 0.05).

**Conclusion:**

Patients with CKD exhibit impaired glymphatic system function, manifested as reduced gBOLD-CSF coupling, which is associated with the severity of CI. These findings support the hypothesis that impaired glymphatic clearance may contribute to cognitive decline in CKD via the kidney-brain axis. Larger longitudinal studies are needed to validate its clinical significance.

## 1 Introduction

Chronic kidney disease (CKD) is a significant public health burden worldwide, with increasing prevalence every year owing to the growing rate of risk factors such as obesity and diabetes (1). An estimated 9% of the global population has CKD of varying severity (2). Patients with CKD not only face systemic complications such as cardiovascular disease, anemia, and electrolyte disorders but are also more susceptible to central nervous system dysfunctions, particularly cognitive impairment (CI). The risk of CI in patients with CKD is significantly higher than that in the general population. Previous studies (3–6) have indicated that the prevalence of CI among patients with CKD ranges from 10 to 40%, which gradually increases with declining kidney function.

However, the specific pathogenesis of CKD-related CI remains incompletely understood. Several hypotheses have been proposed, including cerebral small vessel disease owing to chronic hypoperfusion, inflammation and oxidative stress responses, and cerebral hypoxia caused by anemia (7, 8). However, these mechanisms do not fully explain the high prevalence and specific manifestations of CI in the CKD population. In recent years, the concept of “Kidney-Brain Axis” has been introduced, emphasizing the close pathophysiological interconnection between the kidneys and the brain (7, 9). This connection not only stems from their shared susceptibility to vascular injury (10), but may also involve neurotoxic effects mediated by non-traditional factors such as chronic inflammation, oxidative stress, hypercoagulability, and the accumulation of uremic toxins, ultimately leading to CI (11).

The discovery of the glymphatic system has provided new mechanistic insights into this axial pathway. The glymphatic system, an essential brain clearance pathway discovered in recent years, primarily facilitates the influx of cerebrospinal fluid (CSF) into the perivascular spaces along arteries, driven by astrocytic aquaporin-4 (AQP4) channels. This CSF then mixes with metabolic waste-laden interstitial fluid from the brain parenchyma and is subsequently cleared along perivenous spaces, thereby removing metabolic wastes and maintaining brain homeostasis (12). Previous studies have confirmed that glymphatic system dysfunction is closely associated with the pathogenesis of various neurological disorders, including stroke, Alzheimer’s disease, epilepsy, and idiopathic normal pressure hydrocephalus (13, 14). Recent research indicate that patients with CKD may also have impairment of the glymphatic system (15). Animal experiments have shown that under CKD conditions, the permeability of the blood–brain barrier increases. Uremic toxins induce the activation of matrix metalloproteinase-2, which can disrupt tight junction proteins such as claudin-5, leading to IgG leakage, deposition of insoluble tau protein in the brain parenchyma, and AQP4 polarity disorder, ultimately impairing the brain glymphatic function (16). This suggests that the glymphatic system may act as a critical link in the “Kidney-Brain Axis” in CKD-related CI development, contributing to the imbalance in metabolic toxin clearance and thereby exacerbating brain injury and promoting cognitive decline. Hence, in-depth assessment of the functional status of the glymphatic system using neuroimaging techniques may help explore the neuropathological mechanisms and identify CI biomarkers in patients with CKD.

Recently, studies have explored a non-invasive imaging method to indirectly observe glymphatic activity by decoupling the dynamic temporal processes between the global blood oxygen level-dependent (gBOLD) signal and the CSF inflow signal recorded during resting-state functional magnetic resonance imaging (rs-fMRI) (17, 18). Cardiac pulsation-driven pulsatile expansion of cerebral arteries (i.e., cerebral blood volume and CBV oscillations) compresses the brain parenchyma, thereby promoting the flow of interstitial fluid toward perivenous spaces and driving CSF circulation (19, 20). This process manifests as a specific spatiotemporal coupling relationship between oscillations in the gBOLD signal and oscillations in the CSF inflow signal within the fourth ventricle (21). The strength of this gBOLD-CSF coupling correlates with glymphatic clearance efficiency during sleep (19), AQP4 polarity distribution (22), and cognitive function (23), and has been proposed as a potential neuroimaging biomarker for assessing glymphatic system function in the living human brain.

Therefore, we hypothesize that patients with CKD with CI will exhibit weakened gBOLD-CSF coupling function that is correlated with the degree of CI. In this study, we aimed to validate this hypothesis by enrolling patients with CKD (further subdivided into groups with and without CI) and healthy controls (HCs) and acquiring high-resolution structural MRI and rs-fMRI data. The three specific aims of this study were: (1) to compare gBOLD-CSF coupling between patients with CKD and HCs; (2) to compare this coupling strength between patients with CKD with and without CI; and (3) to explore the correlations of gBOLD-CSF coupling with clinical cognitive scores and laboratory parameters in the CKD cohort. We expected an inverse association between the strength of gBOLD-CSF coupling and CKD and CI.

## 2 Materials and methods

### 2.1 Study design and participants

This study was approved by the Institutional Ethics Committee. All participants provided written informed consent. We retrospectively enrolled patients with CKD and prospectively recruited HCs. The CKD cohort was subsequently categorized into subgroups with and without CI. The diagnosis of CKD was defined as a reduced estimated glomerular filtration rate (eGFR) of > 15 mL/min/1.73 m^2^, which was calculated using the CKD-Epidemiology Collaboration creatinine equation. Participants with CKD had no history of neurological disorders. Similarly, HCs were free of any medical or neurological conditions. Inclusion criteria were as follows: (1) Meeting the diagnostic criteria for CKD; (2) CI indicated by the Montreal Cognitive Assessment—Basic (MoCA) total score: ≤ 19 for 1–6 years of education, ≤ 22 for 7–12 years of education, and ≤ 24 for > 12 years of education; (3) Normal brain MRI findings without significant organic lesions; (4) Aged 18–80 years, regardless of sex; (5) Right-handed; (6) Voluntary participation with signed informed consent. Exclusion criteria were as follows: (1) Patients with CKD or HCs with a history of somatic or neurological disorders; (2) Patients with CKD diagnosed with visual/auditory impairment, traumatic brain injury, acquired immunodeficiency syndrome, or Human Immunodeficiency Virus infection; (3) Individuals with contraindications to MRI scanning; (4) Participants concurrently enrolled in other clinical trials; (5) Individuals with severe physical illnesses who cannot cooperate with MRI examinations. All patients underwent laboratory assessments, including serum urea, creatinine (Cr), uric acid, red blood cell (RBC) count, C-reactive protein, and eGFR. Demographic characteristics and risk factors were recorded, including hypertension (defined as current use of antihypertensive medication or repeated blood pressure measurements >140/90 mmHg) and diabetes mellitus (DM, defined as current use of antidiabetic agents or a confirmed diagnosis at discharge).

A total of 29 patients with CKD (19 with CI and 10 without) and 22 HCs were ultimately included in this study, with ages ranging from 23 to 73 years. The CKD group was significantly older (53.7 ± 15.1 years) than the HC group (43.1 ± 16.0 years, *p* = 0.021), with no significant sex differences.

### 2.2 Image acquisition and preprocessing

All patients with CKD and HCs were scanned using a 3.0 T superconducting MRI scanner (Discovery 750, GE Healthcare, Milwaukee, WI, USA) equipped with a standard 8-channel head phased-array coil in our institution’s radiology department to acquire both functional and structural data. During scanning, foam padding was used to stabilize each participant’s head and minimize motion. After allowing time for acclimatization to the scanner environment, participants were instructed to keep their eyes closed, remain awake, and maintain a relaxed state without engaging in systematic thinking during the MRI acquisition. Routine scanning sequences included localizer images, axial T1-weighted imaging, T2-weighted imaging, and magnetic resonance diffusion-weighted imaging. After excluding intracranial space-occupying lesions and clinically occult diseases on the routine images, high-resolution structural imaging and rs-fMRI scanning were performed. Specifically, T1-weighted structural images were obtained using an isotropic 1 mm^3^ resolution three-dimensional volumetric gradient echo sequence (BRAVO). rs-fMRI was performed using a gradient echo planar imaging sequence, with an in-plane resolution of 3.75 × 3.75 mm (matrix 64 × 64), slice thickness of 5.0 mm, and a repetition time (TR) of 2,000 ms, acquiring a total of 230 continuous volumes.

**Figure 1:**
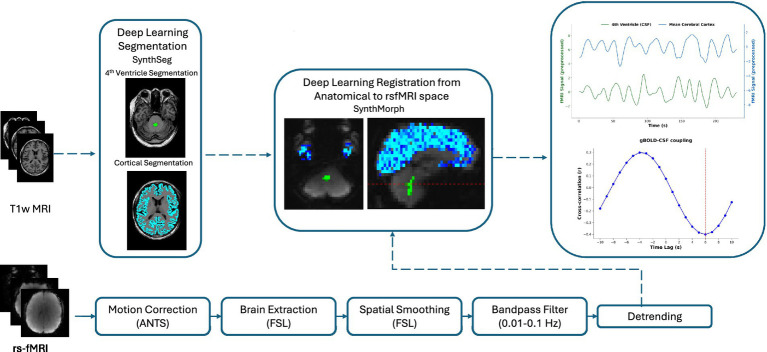
Illustration of the workflow for image procession and fourth ventricle signal extraction and analysis. All rs-fMRI were preprocessed by motion correction, brain extraction, spatial smoothing (4 mm), bandpass filtering (0.01–0.1 Hz), and detrending. Cortical segmentation and fourth ventricle segmentation were performed using SynthSeg on T1-weighted (T1w). Cortical parcellation by FreeSurfer was performed on T1-weighted MRI. All anatomic images, segmented cortical and fourth ventricle spaces were then directly co-registered to brain-extracted images from rs-fMRI data. Co-registered regions were used as masks on preprocessed rs-fMRI data to extract ROIs for gBOLD-CSF coupling analysis. Rs-fMRI, resting-state functional magnetic resonance imaging; ROIs, regions of interest; gBOLD, global blood-oxygen-level-dependent signal; CSF, cerebrospinal fluid.

We used a standardized preprocessing pipeline as illustrated in Figure 1 to preprocess the acquired structural MRI and rs-fMRI data to minimize noise and artifacts and ensure data quality. All preprocessing steps were performed using established neuroimaging software packages and in-house scripts based on Python (version 3.11.0), ensuring the reproducibility of the research results. Briefly, FMRIB Software Library (FSL, version 6.0.7.16) was used for motion correction (MCFLIRT), brain extraction, and spatial smoothing on the rs-fMRI data. For spatial smoothing, a Gaussian kernel with a Full Width at Half Maximum of 4 mm was used. To suppress physiological noise associated with respiratory and cardiac cycles, temporal bandpass filtering was implemented using a two-sided Butterworth filter using the FSL fslmaths tool. The filter passband was set to 0.01–0.1 Hz to effectively isolate low-frequency CSF fluctuations. Additionally, linear detrending was performed on the rs-fMRI time series using NumPy (version 2.2.3) to eliminate signal drift and ensure temporal signal stability for subsequent analysis.

### 2.3 Regions of interest (ROIs) for gBOLD-CSF coupling analysis

Following the workflow shown in Figure 1, we extracted the characteristic CSF signal from the rs-fMRI time series by defining the maximum cross-section of the fourth ventricle as a specific ROI. Specifically, the fourth ventricle was precisely identified (label = 15) from the FreeSurfer aparc + aseg atlas on T1-weighted anatomical images using FreeSurfer’s automated parcellation tool (version 7.4.1), followed by binarization to generate individualized fourth ventricle masks; these masks were directly co-registered to the rs-fMRI space using SynthMorph (24), a contrast invariant deep learning registration method packaged with FreeSurfer, thereby bypassing template-based normalization. This participant-specific approach minimizes potential misregistration errors that may arise from structural variations across individuals. Based on this precise alignment, cross-sectional areas along the *Z*-axis were analyzed to identify the slice with the maximum area as the final ROI for signal extraction.

For gray matter signal extraction, we used a probabilistic tissue segmentation method. The gray matter probability map was thresholded (>0.3) using fslmaths to generate a binary mask. The structural space gray matter mask was co-registered to the fMRI space using the SynthMorph, which employs a deformation field constructed through deep learning to effectively accommodate natural variations in individual brain anatomy. The gBOLD signal was calculated as the temporal average across all voxels within the registered mask. All individual datasets were visually examined by an experienced radiologist to ensure sufficient brain coverage and absence of artifacts, spatial distortion, or missing slices.

### 2.4 Analysis of coupling between gBOLD and CSF signals

**Figure 2:**
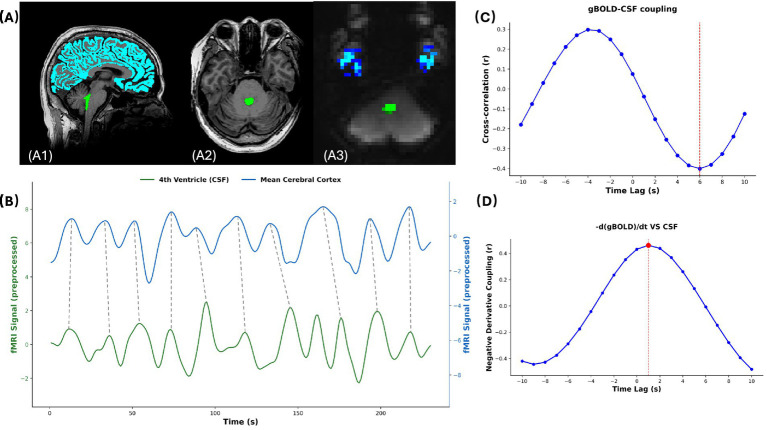
Example of gBOLD-CSF coupling analysis. **(A)** Anatomical localization of signal extraction regions. (A1) Sagittal T1-weighted image showing whole-brain gray matter (blue) and fourth ventricle (green). (A2) Axial T1-weighted image at the level of maximal fourth ventricle cross-section, with the fourth ventricle ROI highlighted in green. (A3) Corresponding resting-state fMRI image aligned with A2, displaying gray matter (blue) and fourth ventricle CSF (green). The regions shown correspond to the final registered masks used for signal extraction following the pipeline in Figure 1. **(B–D)** Coupling analysis results from an example subject. **(B)** Time series of gBOLD (blue) and CSF (green) signals extracted using the registered masks. Dashed vertical lines illustrate the temporal relationship where CSF signal peaks typically occur during the fast-declining period of the gBOLD signal, consistent with BOLD oscillations preceding CSF flow oscillations. **(C)** Cross-correlation analysis between gBOLD and CSF signals across time lags (±10 s). The strongest negative correlation was observed at approximately 6 s (red dashed line), representing gBOLD-CSF coupling. **(D)** Negative derivative coupling analysis validating the CBV oscillation hypothesis. gBOLD, global blood-oxygen-level-dependent signal; CSF, cerebrospinal fluid; ROI, region of interest; CBV, cerebral blood volume.

The gBOLD-CSF coupling was computed using Python (version 3.11.0) on preprocessed rs-fMRI data. The gBOLD signals were extracted from the images before normalization, while the CSF signals were extracted from the fourth ventricle before smoothing, as described in previous studies (17, 21). All signals were first *Z*-normalized and then spatially averaged across all voxels within the ROI. Using NumPy and SciPy libraries (Python 3.11.0), cross-correlations between gBOLD and CSF signals were calculated across a range of time lags (±10 s) to quantify their coupling strength, consistent with prior studies (17). This comprehensive calculation allowed us to observe the complete cross-correlation function. Notably, in our CKD cohort, the time lag of the peak negative correlation varied across individuals. This inter-individual variability aligns with the fact that characteristic lags reported in prior studies also differ across populations [e.g., ~ + 2 s (19), ~ + 3 s and +4 s (17, 21)], suggesting that the optimal delay is not universal. Therefore, to ensure we captured the strongest possible coupling for each participant and to adhere to the physiological principle that BOLD oscillations precede CSF flow, we quantified the gBOLD-CSF coupling strength as the maximum negative correlation coefficient within the 0 to +10 s time-lag window. To demonstrate that oscillations in CBV lead to CSF inflow, we calculated the cross-correlation between the negative derivative of the gBOLD signal and the CSF signal as in previous studies (42). As the coupling index is negative, the opposite value of the gBOLD-CSF coupling was used in subsequent analyses for easier interpretation of the results. Therefore, a larger gBOLD-CSF coupling value reflects a stronger association between global brain activity and CSF flow. A detailed example of this analytical process is provided in Figure 2.

### 2.5 Statistical analyses

Python 3.11.0 was used for statistical analysis. Continuous variables were first assessed for normality using the Shapiro–Wilk test; data conforming to normal distribution are expressed as mean ± standard deviation (SD), whereas non-normally distributed data are expressed as median (interquartile range). Between-group differences in gBOLD-CSF coupling were evaluated using analysis of covariance (ANCOVA) to control for potential confounders. To address potential confounding by demographic factors, we first performed exploratory correlation analyses between gBOLD-CSF coupling and key demographic variables (age, sex, education, hypertension, diabetes) across the entire sample (CKD + HC). Pearson correlation was used for continuous variables (age, education), and point-biserial correlation for binary variables (sex, hypertension, diabetes). False discovery rate (FDR) correction was applied to account for these five demographic comparisons. These analyses were also conducted separately within the CKD and HC groups.

For the comparison between the CKD and HC groups, the primary ANCOVA model included age, sex, years of education, and mean framewise displacement (FD) as covariates. Hypertension and diabetes mellitus status were considered potential mediators on the causal pathway of CKD and were therefore not included in this primary analysis to avoid over-adjustment. To evaluate the robustness of the primary finding against potential confounding by these comorbidities, a sensitivity analysis was performed using an extended model that additionally adjusted for hypertension and diabetes mellitus status; results are presented in Supplementary Figure S1. For the comparison within the CKD cohort (patients with vs. without CI), the ANCOVA model adjusted for age, sex, years of education, hypertension, diabetes mellitus status, and mean FD to control for demographic, clinical, and motion-related confounders. Additionally, to address potential confounding by gBOLD amplitude, the standard deviation of the global BOLD time series was used for supplementary comparison between groups.

To explore the associations between gBOLD-CSF coupling and clinical indicators (MoCA score, eGFR, hemoglobin, red blood cell count, serum urea, serum creatinine, and uric acid), partial correlation analyses were conducted after controlling for age, sex, education, hypertension, and diabetes. This approach allows us to assess the relationship between two variables after removing the linear effects of the specified covariates. To control for false positive results caused by multiple comparisons, the FDR method was applied. Statistical significance was set at a two-tailed *p* < 0.05, and FDR-corrected *p*-values (*q*-values) < 0.05 were considered statistically significant.

## 3 Results

### 3.1 Clinical characteristics of participants

As presented in Tables 1, a total of 29 patients with CKD and 22 HCs were ultimately included in this study. Patients with CKD were significantly older than HCs (53.7 ± 15.1 vs. 43.1 ± 16.0 years, *p* = 0.021). However, no significant differences were observed in sex (male: 58.6% vs. 72.7%, *p* = 0.454) or years of education (*p* = 0.27) between the two groups. The prevalence of hypertension in patients with CKD was significantly higher than that in healthy controls (72.4% vs. 31.8%, *p* = 0.009), whereas the prevalence of diabetes showed no statistically significant difference (31.0% vs. 9.1%, *p* = 0.123).

**Table 1:** Demographic and clinical characteristics of the study participants.

Clinical data	Patients with CKD (*N* = 29)	Healthy controls (*N* = 22)	*p*-value
Age years (SD)	53.7 (15.1)	43.1 (16.0)	0.021
Male, *N* (%)	17 (58.6)	16 (72.7)	0.454
Education, *n* (%)			
Illiterate (0)	4 (13.8)	0 (0)	0.27
Primary school (1)	5 (17.2)	3 (13.6)	
Junior high (2)	9 (31.0)	5 (22.7)	
Senior high (3)	6 (20.7)	4 (18.2)	
College or above (4–5)	5 (17.2)	10 (45.5)	
Diabetes mellitus, *N* (%)	9 (31.0)	2 (9.1)	0.123
Hypertension, *N* (%)	21 (72.4)	7 (31.8)	0.009

Clinical data	CKD patients with CI (*N* = 19)	CKD patients without CI (*N* = 10)	*p*-value
Age years (SD)	58.3 (13.9)	44.9 (12.6)	0.019
Male, *N* (%)	12 (63.2)	5 (50.0)	0.694
Education, *n* (%)			
Illiterate (0)	3 (15.8%)	0 (0.0%)	0.000
Primary school (1)	4 (21.1%)	1 (10.0%)	
Junior high (2)	8 (42.1%)	3 (30.0%)	
Senior high (3)	3 (15.8%)	5 (50.0%)	
College or above (4–5)	1 (5.3%)	1 (10.0%)	
Diabetes mellitus, *N* (%)	7 (36.8%)	2 (20.0%)	0.431
Hypertension, *N* (%)	13 (68.4%)	8 (80.0%)	0.675
Cr (μmol/L), median (IQR)	140.0 (106.0–214.5)	124.5 (109.5–220.8)	0.891
Serum urea (mmol/L), median (IQR)	10.2 (6.7–13.0)	8.0 (6.3–11.4)	0.449
RBC (×10^12^/L), mean ± SD	9.5 ± 23.2	4.0 ± 0.6	0.598
Hb (g/L), mean ± SD	114.6 ± 19.8	123.9 ± 21.1	0.256
eGFR, median (IQR)	33.4 (18.3–49.2)	38.8 (25.1–49.0)	0.536
Uric acid (μmol/L), median (IQR)	322.0 (292.0–411.0)	343.5 (299.8–394.0)	0.872
MoCA score, mean ± SD	21.6 ± 2.7	27.8 ± 1.2	0.000

Data are presented as mean ± standard deviation, median (interquartile range), or number (%). Education was coded as: Illiterate (0), Primary school (1), Junior high (2), Senior high (3), College or above (4–5). CKD, chronic kidney disease; CI, cognitive impairment; eGFR, estimated glomerular filtration rate; MoCA, Montreal Cognitive Assessment. *p*-values are for intergroup comparisons.

Among patients with CKD, those with CI (*n* = 19) were significantly older than those without CI (*n* = 10) (58.3 ± 13.9 vs. 44.9 ± 12.6 years, *p* = 0.019) and had fewer years of education (*p* < 0.001). However, no significant differences were observed between the two groups in terms of sex, diabetes, hypertension, renal function parameters (serum creatinine, urea, eGFR, uric acid), or RBC count (all *p* > 0.05).

### 3.2 Differences in gBOLD-CSF coupling between the groups

**Figure 3:**
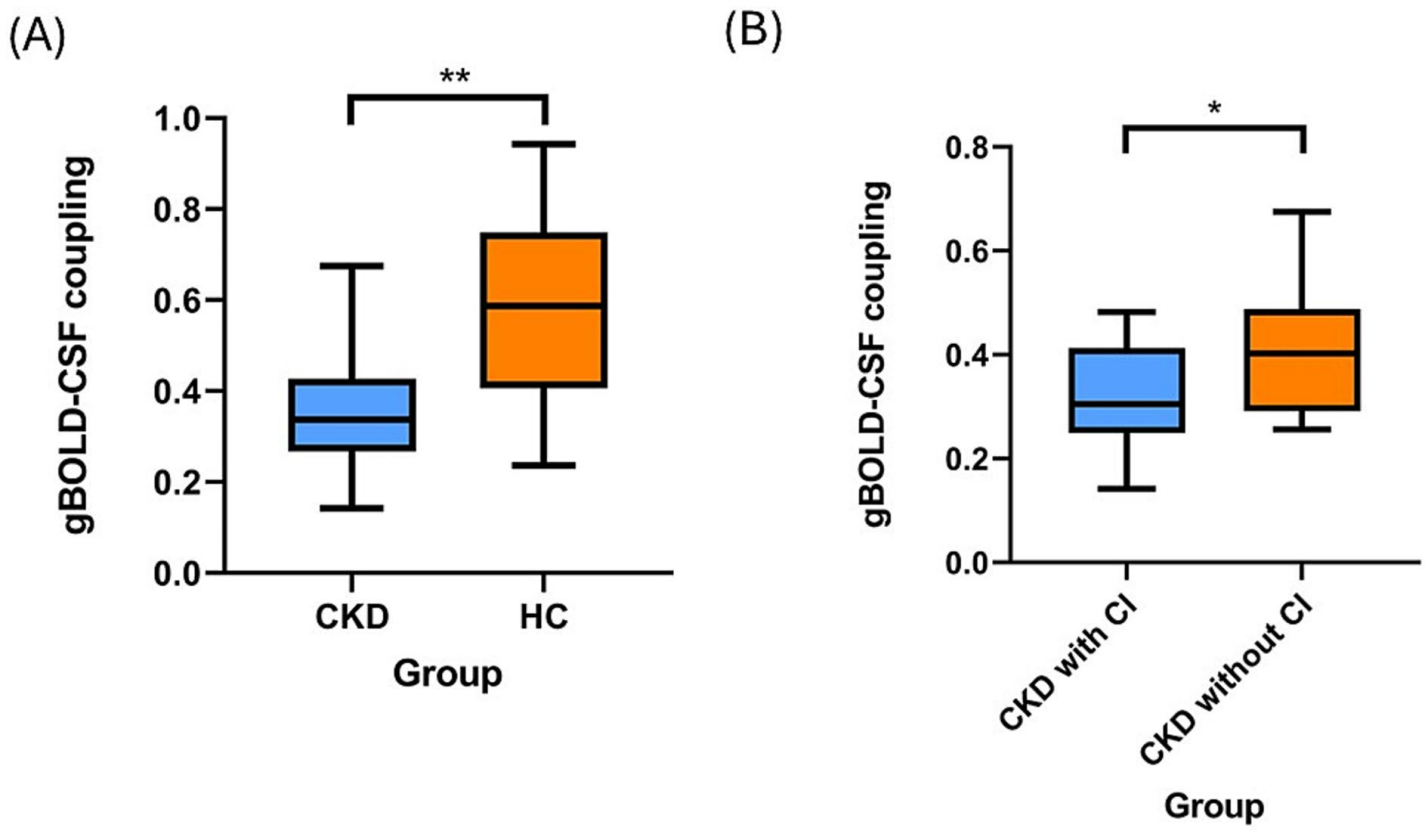
Differences in gBOLD-CSF coupling between the groups. The primary ANCOVA, adjusted for age, sex, education, and mean framewise displacement (FD), revealed that patients with CKD exhibited significantly lower gBOLD-CSF coupling compared to HCs (*β* = −0.178, 95% CI: −0.291 to −0.066, *p* = 0.003) **(A)**. After adjusting for age, sex, education, hypertension, diabetes mellitus status, and FD, patients with CKD with CI showed significantly lower gBOLD-CSF coupling than those without CI (*β* = −0.135, 95% CI: −0.263 to −0.007, *p* = 0.040) **(B)**. A box is drawn from the 25th to 75th percentiles, and a horizontal line is drawn at the median. gBOLD-CSF, global blood-oxygen-level-dependent to cerebrospinal fluid; CKD, chronic kidney disease; HCs, healthy controls; CI, cognitive impairment; CSF, cerebrospinal fluid. * means *p* < 0.05 and ** means *p* < 0.01.

The primary ANCOVA revealed that patients with CKD exhibited significantly lower gBOLD-CSF coupling compared to HCs (*β* = −0.178, 95% CI: −0.291 to −0.066, *p* = 0.003; Figure 3A). This finding remained robust in a sensitivity analysis that further adjusted for hypertension and diabetes mellitus status (*β* = −0.195, 95% CI: −0.313 to −0.076, *p* = 0.002; Supplementary Figure S1), with neither comorbidity showing significant independent effects (hypertension: *p* = 0.717; diabetes: *p* = 0.177). Within the CKD cohort, patients with CI showed significantly lower gBOLD-CSF coupling than those without CI (*β* = −0.135, 95% CI: −0.263 to −0.007, *p* = 0.040; Figure 3B).

### 3.3 Correlation analysis of the association between gBOLD-CSF coupling and clinical characteristics

We first examined the associations between gBOLD-CSF coupling and key demographic variables across all participants (CKD and HC). In unadjusted analyses, coupling strength showed a negative correlation with age (*r* = −0.345, *p* = 0.013) and a positive correlation with education level (*r* = 0.304, *p* = 0.030). However, after FDR correction for these five demographic comparisons (age, sex, education, hypertension, diabetes), neither correlation remained statistically significant (both *q* > 0.05). No significant correlations were observed with sex, hypertension, or diabetes status (all *p* > 0.05). Importantly, within the CKD group alone, none of these demographic variables showed a significant correlation with gBOLD-CSF coupling (all *p* > 0.25; see Supplementary Table S1 for full results including correlation coefficients, 95% confidence intervals, and FDR-corrected *q*-values). These results justify the inclusion of age, sex, and education as covariates in our primary ANCOVA models.

**Figure 4:**
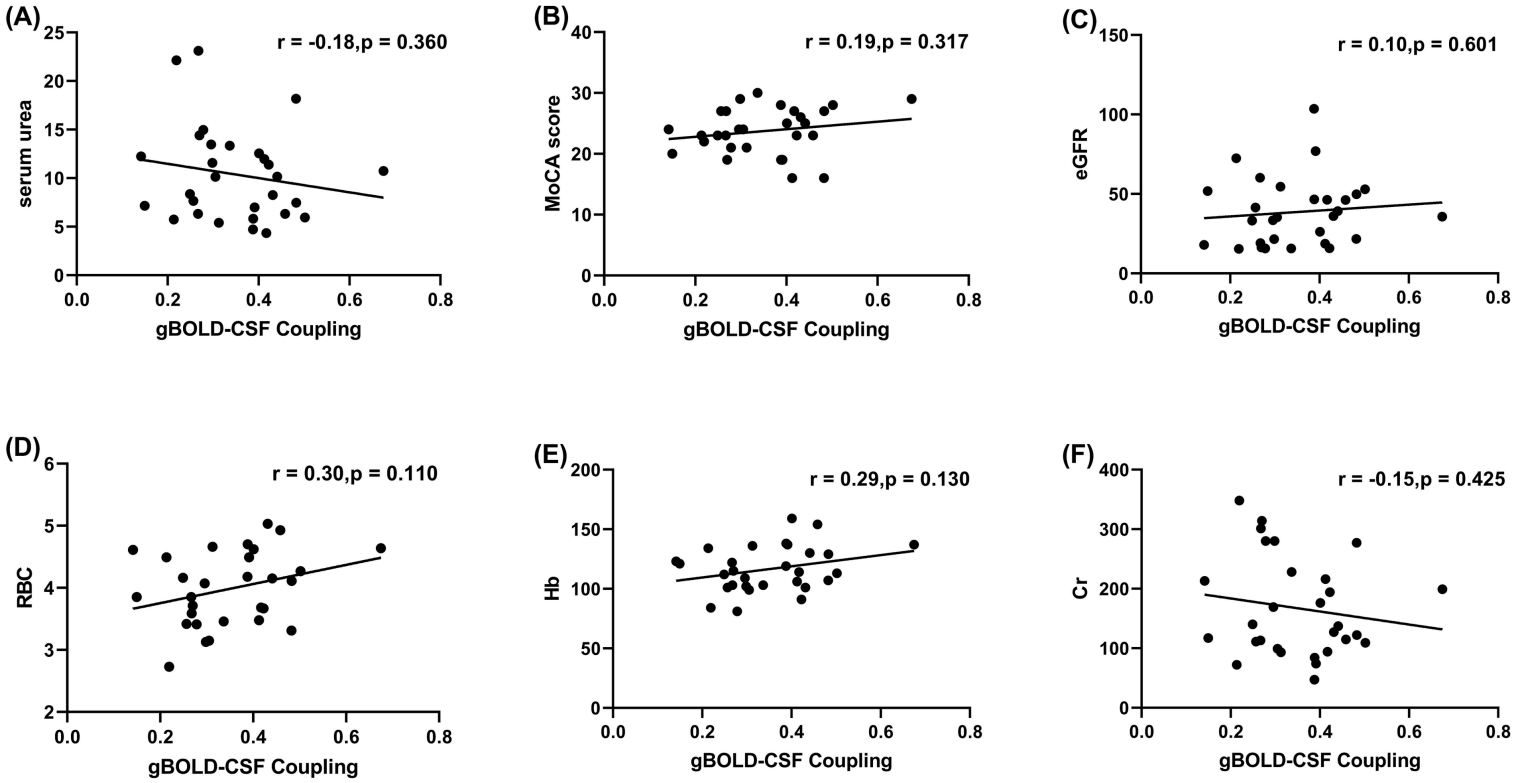
Scatter plots showing partial correlations between gBOLD-CSF coupling and clinical parameters, after controlling for age, sex, education, hypertension, and diabetes. **(A)** Correlation between gBOLD-CSF coupling and serum urea (mg/dL); **(B)** Correlation between gBOLD-CSF coupling and MoCA score; **(C)** Correlation between gBOLD-CSF coupling and eGFR (mL/min/1.73 m^2^); **(D)** Correlation between gBOLD-CSF coupling and RBC (×10^12^/L); **(E)** Correlation between gBOLD-CSF coupling and Hb (g/dL); **(F)** Correlation between gBOLD-CSF coupling and serum creatinine (mg/dL). No statistically significant correlations were observed between gBOLD-CSF coupling and any of the tested parameters after FDR correction (all *q* > 0.05). Prior to FDR correction, weak correlation trends were noted: positive correlations with hemoglobin (*r* = 0.29, *p* = 0.13), MoCA score (*r* = 0.19, *p* = 0.317), red blood cell count (*r* = 0.30, *p* = 0.110), and eGFR (*r* = 0.10, *p* = 0.601); negative correlations with serum urea (*r* = −0.18, *p* = 0.36) and serum creatinine (*r* = −0.154, *p* = 0.425). None of these associations were statistically significant. gBOLD, Global Blood-Oxygen-Level-Dependent signal; CSF, cerebrospinal fluid; MoCA, Montreal Cognitive Assessment; eGFR, estimated glomerular filtration rate; Hb, hemoglobin; RBC, red blood cell count; Cr, creatinine; FDR, false discovery rate.

Next, partial correlation analysis, after controlling for age, sex, education, hypertension, and diabetes, was performed to examine the relationships between gBOLD-CSF coupling and clinical indicators (including serum uric acid, MoCA score, eGFR, serum creatinine, red blood cell count, and hemoglobin). After FDR correction, no statistically significant correlations were observed (all *q* > 0.05). The uncorrected correlation coefficients, scatter plots for which are presented in Figure 4, were all of small effect size and non-significant. Therefore, no robust associations were found between gBOLD-CSF coupling and the assessed clinical parameters in this cohort. A supplementary analysis showed no significant difference in gBOLD amplitude between CKD patients and HCs after adjusting for age, sex, and education (*β* = 1.783, *p* = 0.095).

## 4 Discussion

The results of this study demonstrate that gBOLD-CSF coupling in patients with CKD is lower than that in HCs, which may be related to glymphatic dysfunction. Moreover, within the CKD group, those with CI had significantly lower gBOLD-CSF coupling than those without CI, suggesting that glymphatic system dysfunction may be associated with CKD-related cognitive decline. Furthermore, although we investigated the relationship between gBOLD-CSF coupling and clinical indicators (such as eGFR, creatinine, and MoCA scores), no statistically significant correlations were observed after multiple comparisons correction. These findings provide important evidence for understanding the mechanisms underlying CI in patients with CKD.

As we know, CSF constitutes a key component of the glymphatic system. Although previous studies have predominantly used phase-contrast MRI (PC-MRI) to assess CSF flow (25, 26), PC-MRI has relatively low temporal resolution, making it difficult to capture the faster physiological oscillations closely related to neural activity. Based on the mechanism proposed by Fultz et al. (19)—whereby neural slow-wave oscillations induce rhythmic changes in CBV—under the constraint of a fixed intracranial volume, a reduction in CBV creates inflow space for CSF and drives its large-scale flow.

We utilized rs-fMRI data to evaluate glymphatic system function by analyzing the coupling between global BOLD signals and CSF signals in the fourth ventricle (gBOLD-CSF coupling). The fourth ventricle was chosen as the ROI because, as a key hub for CSF circulation, it is adjacent to the brainstem and cerebellar structures, potentially making it more sensitive to changes in central nervous system fluid dynamics. Meanwhile, CSF signals from the fourth ventricle were obtained by automatically segmenting the fourth ventricle using FreeSurfer and registering it to functional space, ensuring the accuracy and reproducibility of signal extraction. Recently, this gBOLD-CSF coupling has been confirmed by multiple studies as an effective non-invasive biomarker for assessing glymphatic system function. It has been consistently shown to be abnormally reduced in neurodegenerative disorders such as Alzheimer’s disease and Parkinson’s disease, as well as in psychiatric conditions including generalized anxiety disorder and early-stage psychosis. Furthermore, this reduction is closely associated with CI (17, 21, 27–29). Notably, this coupling still exists in the awake resting state and is highly reliable (18, 28), providing key evidence for non-invasive research. This suggests that glymphatic system dysfunction may represent a common pathway underlying CI across diverse disease categories—from psychiatric disorders to neurological conditions. Our findings in patients with CKD further support and extend this hypothesis. Notably, the observed reduction in gBOLD-CSF coupling in CKD patients persisted in sensitivity analyses adjusting for hypertension and diabetes (Supplementary Figure S1), suggesting that this glymphatic impairment is not merely attributable to these common comorbidities.

To our knowledge, this study first confirmed that patients with CKD have reduced gBOLD-CSF coupling, which provides novel and important neuroimaging evidence for the “kidney-brain axis” concept. The gBOLD-CSF coupling is considered to reflect the driving force of cardiac pulsation-induced cerebral arterial pulsatile expansion (CBV oscillations) on CSF flow (30–32). Weakened gBOLD-CSF coupling may indicate impairment of this driving process, which compromises the metabolic waste clearance efficiency of the glymphatic system (33). This finding is consistent with those of previous animal studies: CKD models have demonstrated impaired blood–brain barrier integrity, disrupted AQP4 polarity, and accumulation of toxic substances such as tau protein in the brain (16). Our results suggest that similar pathophysiological processes are probably present in patients with CKD. Declining kidney function leads to the accumulation of uremic toxins (such as indoxyl sulfate), which further disrupts blood–brain barrier integrity. This process, mediated through the activation of inflammatory pathways (such as nuclear factor kappa B) and oxidative stress, results in the loss of astrocytic AQP4 polarity, thereby impairing glymphatic clearance efficiency (9, 34–36). Furthermore, CKD-related vascular pathologies (such as microvascular endothelial dysfunction and arteriosclerosis) may weaken arterial pulsatility, which is a key driving force for CSF flow (37). These changes collectively lead to the accumulation of metabolic waste products (such as *β*-amyloid and tau proteins) in the brain parenchyma, triggering neuroinflammation and neuronal damage, and ultimately promoting the development of CI (38, 39).

Notably, we found that the impairment in gBOLD-CSF coupling was more severe in the CKD subgroup with CI. This indicates that glymphatic system dysfunction is not a common accompaniment of CKD, but may be closely related to cognitive status.

Although the direct correlation between gBOLD-CSF coupling and MoCA scores did not reach statistical significance, the significant between-group differences strongly suggest that reduced glymphatic clearance efficiency is an important contributing factor to CI in patients with CKD. This opens a new perspective for understanding the mechanisms of CKD-related CI: in addition to traditional factors such as cerebrovascular disease and anemia, failure of the brain’s clearance system may lead to abnormal accumulation of neurotoxic substances (such as *β*-amyloid and tau proteins), thereby accelerating neurodegeneration and cognitive decline (40, 41).

The absence of statistically significant correlations between gBOLD-CSF coupling and clinical parameters such as eGFR, MoCA scores, and hemoglobin after multiple comparisons correction warrants discussion. This null finding could be attributed to several factors, with the limited sample size of this exploratory study being a primary consideration, as it inherently restricts statistical power to detect small-to-moderate effects. Furthermore, the relationship between glymphatic function and systemic clinical markers in CKD is likely complex and multifactorial, potentially mediated by intermediary pathophysiological processes not directly captured by these blood-based measures. Therefore, the lack of robust linear associations in our data does not preclude the existence of more complex, non-linear, or indirect relationships, which should be investigated in larger, hypothesis-driven cohorts.

Furthermore, our exploratory analysis of demographic factors revealed that gBOLD-CSF coupling showed trends of association with age and education in the entire sample, though these did not survive multiple comparison correction. The lack of significant correlations within the CKD group specifically suggests that the observed reduction in coupling among CKD patients is not merely a reflection of these common demographic factors, but rather a characteristic related to the disease state itself.

### 4.1 Limitations

This study has certain limitations. First, the statistical power may be inadequate as it is a single-center, cross-sectional study with a small and limited sample size; this is possibly the primary reason why the correlations between gBOLD-CSF coupling and clinical indicators such as hemoglobin, MoCA score, and eGFR only showed consistent trends without reaching statistical significance.

Second, although routine MRI scans of all enrolled patients did not show obvious structural lesions, the absence of more sensitive sequences (such as SWI) indicates that clinically asymptomatic cerebral microbleeds, which could affect cognitive function, could not be ruled out. These occult cerebrovascular lesions may act as confounding factors. Finally, the specificity of the gBOLD-CSF coupling analysis used in this study requires further validation in future research. Overall, this study serves as a pilot investigation that preliminarily confirms the presence of imaging evidence of glymphatic system dysfunction in patients with CKD, particularly those with CI.

## 5 Conclusion

This study indicates that patients with CKD have significant impairment of the brain’s glymphatic system, as evidenced by markedly reduced gBOLD-CSF coupling compared to HCs. Notably, this dysfunction was more severe in patients with CKD with CI. Our study provides novel neuroimaging evidence supporting the “kidney-brain axis” paradigm and suggesting that compromised glymphatic clearance may contribute to cognitive decline in CKD. Although the gBOLD-CSF coupling did not show statistically significant correlations with conventional clinical indicators such as eGFR and MoCA scores, its consistent directional trends are indicatory. Future longitudinal studies with larger samples are required to elucidate the causal relationship and explore the potential of its clinical application.

## Data Availability

The raw data supporting the conclusions of this article will be made available by the authors, without undue reservation.
