# Supplementary figures and images for: Reduced global BOLD-CSF coupling in chronic kidney disease-related cognitive impairment: a resting-state functional MRI study
Source: Front Neurol. 2026 Jan 12;16:1738198. doi: 10.3389/fneur.2025.1738198 (PMC12832948; doi:10.3389/fneur.2025.1738198)

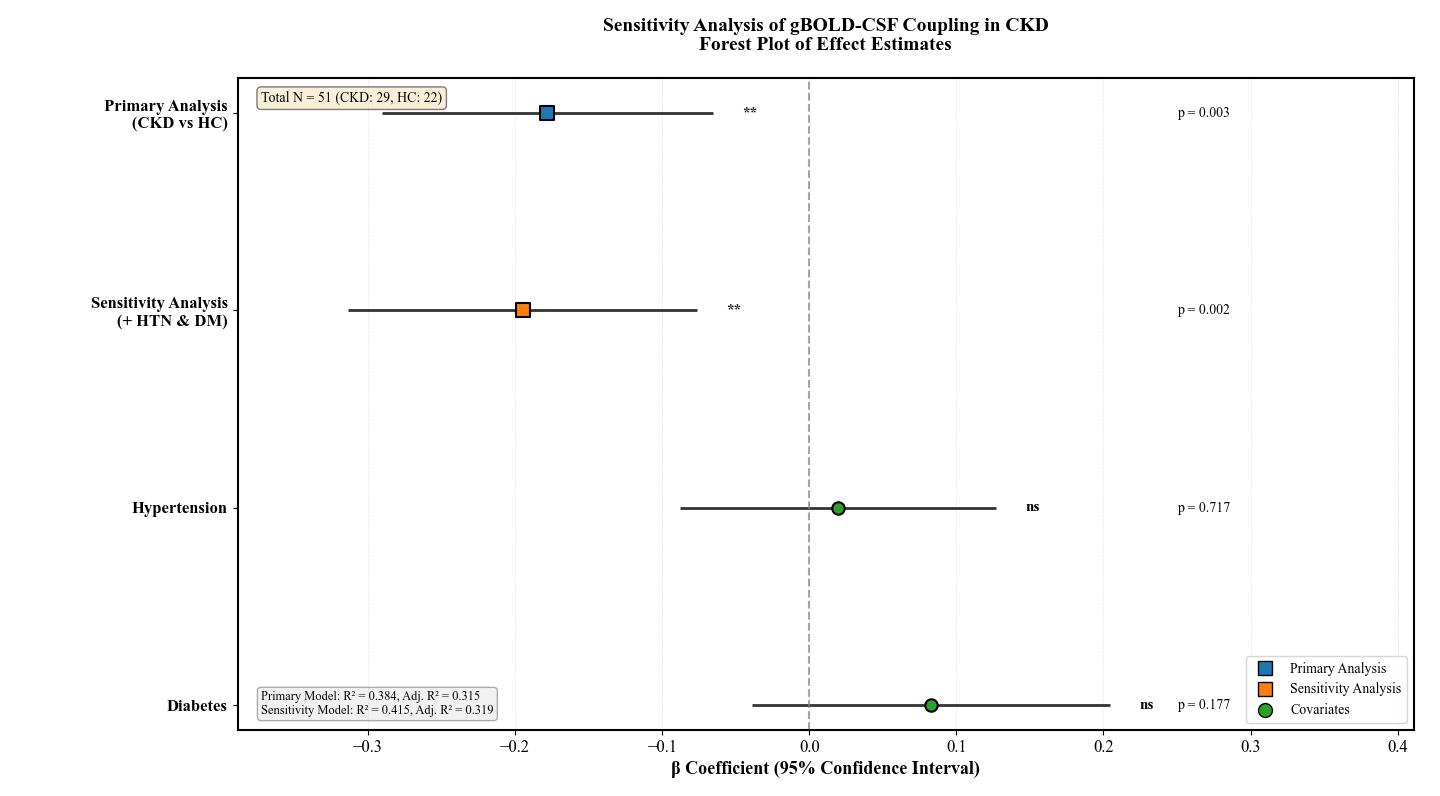

Supplement: Supplementary file 1 [file Image_1.png]
