# Supplementary material for: Reduced global BOLD-CSF coupling in chronic kidney disease-related cognitive impairment: a resting-state functional MRI study
Source: Front Neurol. 2026 Jan 12;16:1738198. doi: 10.3389/fneur.2025.1738198 (PMC12832948; doi:10.3389/fneur.2025.1738198)
Supplement: Supplementary file 2 [file Table_1.docx]

**Supplementary Material**

**Supplementary Figure 1.** Sensitivity analysis forest plot for gBOLD-CSF coupling in CKD

Effect estimates from primary (blue square) and sensitivity ANCOVA models (orange square) are compared. The primary model adjusts for demographics and head motion; the sensitivity model additionally adjusts for hypertension and diabetes. Green circles represent comorbidity-specific effects. Error bars denote 95% CIs. The significant reduction in coupling among CKD patients remained after comorbidity adjustment (β = –0.195, p = 0.002). Neither hypertension (β = 0.019, p = 0.717) nor diabetes (β = 0.083, p = 0.177) exerted significant independent effects.

**Supplementary Table S1** Correlations between gBOLD-CSF coupling and demographic variables

| Variable | Sample | n | Correlation Coefficient (r) | 95% CI for r | Uncorrected p-value | FDR-corrected q-value |
| --- | --- | --- | --- | --- | --- | --- |
| Age | Entire Sample | 51 | -0.345 | -0.567, -0.077 | 0.013 | 0.066 |
|  | CKD Only | 29 | -0.221 | -0.549, 0.176 | 0.250 | 0.500 |
| Education | Entire Sample | 51 | 0.304 | 0.031, 0.535 | 0.030 | 0.075 |
|  | CKD Only | 29 | 0.160 | -0.221, 0.503 | 0.407 | 0.678 |
| Sex (Male) | Entire Sample | 51 | 0.087 | -0.193, 0.354 | 0.544 | 0.679 |
|  | CKD Only | 29 | 0.097 | -0.290, 0.458 | 0.615 | 0.769 |
| Hypertension | Entire Sample | 51 | -0.204 | -0.454, 0.076 | 0.152 | 0.253 |
|  | CKD Only | 29 | -0.138 | -0.488, 0.249 | 0.475 | 0.678 |
| Diabetes mellitus | Entire Sample | 51 | -0.036 | -0.308, 0.242 | 0.804 | 0.804 |
|  | CKD Only | 29 | 0.087 | -0.300, 0.451 | 0.655 | 0.819 |

The table shows correlation coefficients (r), uncorrected p-values, false discovery rate (FDR)-corrected q-values, and 95% confidence intervals (CI) for the associations between gBOLD-CSF coupling and demographic variables. Analyses were performed for the entire sample (Patients with CKD and Healthy Controls combined) and separately for the CKD group. Pearson correlation was used for continuous variables (Age, Education); point-biserial correlation was used for binary variables (Sex [Male=1, Female=0], Hypertension [Yes=1, No=0], Diabetes [Yes=1, No=0]). CI = confidence interval; FDR = false discovery rate.
